# An assessment of serum leptin levels in patients with chronic viral hepatitis: a prospective study

**DOI:** 10.1186/1471-230X-7-17

**Published:** 2007-05-31

**Authors:** Spilios Manolakopoulos, Sotirios Bethanis, Charis Liapi, Fotini Stripeli, Pantelis Sklavos, Alexandra Margeli, Aggeliki Christidou, Aggeliki Katsanika, Evangellos Vogiatzakis, Dimitrios Tzourmakliotis, Stamatios Theocharis

**Affiliations:** 1 Department of Gastroenterology, Polyclinic General Hospital, Athens, Greece; 2 Department of Pharmacology, University of Athens, School of Medicine, Athens, Greece; 3 Department of Forensic Medicine and Toxicology, University of Athens, School of Medicine, Athens, Greece; 4 Department of Microbiology, Polyclinic General Hospital, Athens, Greece

## Abstract

**Background:**

The role of leptin in the course of liver disease due to chronic viral hepatitis (CVH) remains controversial. Our aims were to investigate the relationship between serum leptin concentrations and the severity of liver disease in a cohort of subjects with HBeAg negative chronic hepatitis B (CHB) and C (CHC) and to analyze the effect of body composition, the leptin system and insulin resistance together with viral factors on virologic response to antiviral treatment.

**Methods:**

We studied 50 (36 men) consecutive patients suffering from biopsy-proven CVH due to HBV (n = 25) or HCV (n = 25) infection. Thirty-two (17 men) healthy volunteers served as controls. Levels of serum leptin and insulin were determined by immunoassays at baseline and at the end of the treatment.

**Results:**

A significant association between serum leptin levels and the stage of hepatic fibrosis was noted; patients with cirrhosis presented higher serum leptin levels compared to those with lower fibrosis stage [CHB patients (17436 pg/ml vs 6028.5 pg/ml, p = 0.03), CHC patients (18014 pg/ml vs 4385 pg/ml, p = 0.05]. An inverse correlation between lower leptin levels and response to lamivudine monotherapy was noted in patients with CHB; those with a virologic response presented lower serum leptin levels (5334 vs 13111.5 pg/ml; p-value = 0.003) than non-responders. In genotype 1 CHC patients, insulin resistance played a significant role in the response to antiviral therapy.

**Conclusion:**

Our data clearly suggest that cirrhosis due to CHB or CHC is associated with higher leptin levels. Increased serum leptin levels represent a negative prognostic factor for response to lamivudine monotherapy in patients with CHB. In CHC patients insulin resistance strongly influences the response to antiviral treatment in patients infected with genotype 1.

## Background

Leptin is a circulating 16-kDa non-glycosylated protein secreted from the adipocytes of white fat into the blood [1]. The levels of leptin are related to the adipose tissue mass, and signal the central nervous system of the presence of sufficient energy stores, causing a response characterized by anorexia and increased energy expenditure [2].

Leptin has been also implicated in many actions including liver fibrogenesis [3-6]. Serum leptin levels have been found higher in patients with chronic hepatitis C (CHC) and particular in those with more severe fibrosis or cirrhosis [7]; however the results are conflicting [8-14].

Recent observations in patients with CHC indicate that liver steatosis and insulin resistance has been associated with progressive hepatic fibrosis and sustained virologic response (SVR) to antiviral treatment [15]. It is known that serum leptin regulates insulin secretion and tissue responsiveness to this hormone [16]. Nevertheless, the relationship between leptin and insulin resistance in patients with chronic viral hepatitis (CVH) seems obscure at present; both presence [12] and absence [13] of association between serum leptin and insulin levels have been observed in CVH patients.

In view of the obscure role of serum leptin in CVH we were interested in its role in chronic hepatitis B (CHB) and CHC infection. To investigate this we measured the serum leptin levels in 25 patients with HBeAg negative CHB and 25 patients with CHC before and after antiviral therapy and matched controls and assessed its relationship to anthropometric, metabolic and histopathological parameters. The aims of the present study were (a) to assess the relationship between serum leptin levels and the stage of liver disease in a cohort of subjects with CHB and CHC, (b) to evaluate the relationship among leptin, insulin and the stage of hepatic fibrosis and (c) to evaluate the effect of body composition, the leptin system and insulin resistance together with viral factors on SVR in patients with CVH receiving antiviral therapy.

## Methods

### 2.1 Patients

This prospective open-label study included 50 consecutive patients with hepatitis C virus (HCV) (n = 25) or hepatitis B virus (HBV) (n = 25) infection referred to our Liver Unit between January 2004 and January 2006. All patients were evaluated with physical examination, laboratory tests and a baseline liver biopsy. Entry criteria included patients aged 16–75 year, elevated alanine aminotransferase (ALT) levels for at least 6 months on at least 3 occasions and HBsAg or anti-HCV and HCV-RNA positivity. We excluded patients with: (a) Other causes of chronic liver disease (HBsAg for HCV patients, anti-HCV or detectable antibodies against hepatitis delta for HBV patients, alcoholism, Wilson's disease, drugs, haemochromatosis, autoimmune hepatitis), (b) Seropositivity for anti-HIV, (c) Decompensated liver disease, (d) History of heart failure, diabetes, thyroid diseases, abnormal renal function and cancer, (e) Morbid obesity i.e. body mass index (BMI) > 40, (f) Contraindications or previous treatment with antiviral agents (interferon, nucleos(t)ide analogues), metformin, vitamin E, or a thiazolidinedione and (g) Use of drugs known to induce liver steatosis (corticosteroids, amiodarone, tamoxifen, valproic acid) within the last 6 months. Weight alterations over the previous year were recorded, and patients with changes more than 10% were also excluded from the study.

### 2.2 Controls

The control group comprised 32 (17 men and 15 women) healthy volunteers (doctors, nurses, students) matched for BMI from Polykliniki General Hospital and considered healthy on the basis of history, physical examination and laboratory tests. None received any medication and had normal liver enzymes and no clinical, laboratory or imaging evidence of liver disease. Subjects who have had a restriction of diet for losing weight during the last three months have not been included into control group. The study was performed in accordance with the Helsinki Declaration II, with the approval of the Ethics Committee of Polyclinic General Hospital and all patients and control subjects gave their verbal informed consent.

### 2.3 Clinic and Laboratory Evaluation

Clinical and laboratory data were collected on the date that baseline liver biopsy was performed. A complete medical history and physical examination was accomplished in all patients and controls. Height (cm) and weight (kg) were determined at baseline to calculate BMI, as an expression of the overall state of nutrition. The fat mass index (FMI) was calculated according to the formula FMI = (BMI-13)/1.33. Blood samples were obtained at 08.00–09.00 h after an overnight fast, stored for one hour at room temperature, centrifuged and serum was separated. Biochemical analyses were performed at the same day. Concentrations of glucose, ALT, aspartate aminotransferase (AST), total bilirubin, albumin, total cholesterol and triglycerides were measured. Commercially available enzyme immunoassays (EIA) were used for the detection of HBsAg, HbeAg, anti-HBe, anti-HBc (Roche Diagnostics GmbH, D-68298 Mannheim). Anti-HCV was tested by a third-generation EIA (Roche Diagnostics GmbH, D-68298 Mannheim). Serum HBV DNA concentrations were determined by a commercially available quantitative assay (Amplicor HBV MONITOR assay, Roche Diagnostics, GmbH Mannheim, Germany). Serum HCV RNA determination was carried out by the Amplicor HCV test (Roche Molecular System). HCV genotyping was performed by reverse hybridization (InnoLiPA HCV; Innogenetics, Gent, Belgium).

Insulin was measured using a two-site electrochemiluminescence immunoassay performed on the Roche Elecsus immunoassay analyzer. Intra-assay C.V.'s were 2.6, 2.8 and 2.5% at 6.36, 20.9,747 μU/mL, respectively.

Serum samples for measurement of leptin were stored at -80°C until subsequent analysis. The quantitative determination of human leptin was conducted in serum by solid-phase ELISA techniques, using commercially available kit, purchased from R&D Systems Inc., (Minneapolis, USA). Every sample was run in duplicate, measurements differed by less than 10%, and the mean value was calculated and used for statistical analysis. The intra- and inter-assay precision coefficient of variation (CV %) of leptin ranged between 3.0–3.3 and 3.5–5.4 % respectively at different levels. Serum glucose and insulin levels were used to determine the homeostatic model assessment (HOMA-IR) described by Matthews et al using the formula IR = (insulinxglucose)/22.5 [17]. Higher insulin resistance is indicated by higher values of HOMA-IR. As previously recommended, insulin resistance was arbitrarily considered when it was > 2, whereas HOMA-IR values > 4 were considered as a prediabetic state [18].

### 2.4 Study Protocol

All HBV patients were treated with orally lamivudine 100 mg daily. HCV patients received 180 μg of peginterferon alfa-2α weekly, plus ribavirin according to genotype and body weight [19]. Onset of treatment was not over 2 months after baseline liver biopsy. All patients were followed up in the out-patient clinic and had physical examination and blood drawn for liver chemistry, creatinine, full blood count prothrombin time, cholesterol and triglyceride at months 1, 2, 3 and then every 3 months for HBV patients and every month for HCV patients. Serum HBV DNA and HCV RNA were evaluated at least every 3–6 months. Serum leptin and insulin were measured at last visit for HBV patients and 3–6 months after treatment discontinuation for HCV patients.

Virological response was considered as the clearance of serum HBV DNA by polymerase chain reaction (PCR) assay and biochemical response as the decline of transaminase levels within normal range. Virological breakthrough was considered as the reappearance of detectable serum HBV DNA by PCR after initial response. For patients with chronic HCV infection SVR was considered when HCV RNA was not detected by qualitative PCR 6 months after treatment discontinuation.

### 2.5 Histological Assessment

All patients underwent baseline liver biopsy; 36 underwent a follow-up liver biopsy (23 with CHB at the end of the 1^st ^year of treatment and 13 with CHC at or about the end of treatment). Liver biopsies were evaluated by experienced pathologists who were unaware of the clinical and virological status of patients. The histologic lesions were analyzed according to Ishak classification system [20]. Steatosis was assessed as the percentage of hepatocytes containing macrovesicular fat droplets. It was graded as follows: 0 = no steatosis; 1 = mild: < 33% of hepatocytes affected; 2 = moderate: 33% to 66% of hepatocytes affected; 3 = severe: > 66% of hepatocytes affected [21].

### 2.6 Statistical Analysis

Analyses were performed using STATA 8.0 statistical package (Stata Corporation, College Station, TX, U.S.A.). The clinical, biochemical, and virologic data are presented as median with range as most data did not show a Gaussian distribution. Serum HBV DNA and HCV RNA concentrations below detection limit (< 400 copies/mL and 50IU/l or 260 copies/mL respectively) were imputed to 399 copies/mL and 259 copies/mL respectively for the analyses. All HBV DNA and HCV RNA values were log-transformed for normality. Continuous variables were compared using the Wilcoxon's rank-sum and Mann-Whitney tests. Categorical variables were compared using χ^2 ^or Fisher's exact tests as appropriate. Spearman's correlation coefficients were calculated for estimation of the level of association between two variables. A multiple regression analysis was performed with leptin as the dependent variable and age, gender, BMI, FMI, histological and biochemical data as independent variables. Two-sided p-values less than 0.05 were considered statistically significant.

## Results

### 3.1 Characteristics of the patient and control population

Due to the gender dependency of serum leptin levels, patients and controls were divided in two groups (men and women-Table 1). All patients with HBV infection were HBeAg (-), anti HBeAg (+) and the median serum HBV DNA concentrations were 784000 cp/mL (770–192000000). The median serum HCV RNA concentrations were 769000 cp/mL (400, 2452635). Genotype distribution in patients with HCV hepatitis was as follows: 11 patients (44%) were infected with genotype 1, 9 (36%) with genotype 3 and 5 (20%) with genotype 4. Anthropometric, metabolic and histological parameters were similar in CHB and in CHC patients.

Table 2 summarizes the liver histology findings. Severe steatosis was observed only in one CHC patient. The percentage of steatosis was not different between CHB and CHC group (p = 0.5) and between cirrhotics and non-cirrhotics (p = 0.7).

### 3.2 Relationship between serum leptin and insulin levels and clinical, biochemical and histological data of patients and controls

Serum leptin levels were significantly higher in females than in males in CHB (p = 0.05), CHC (p = 0.05), cirrhotic patients' group (p = 0.04) and also the control group (p < 0.001).

Non-cirrhotic female patients both in CHB and CHC patients' group presented significantly lower serum leptin levels than controls (p = 0.01). Patients with CHB and definite histological diagnosis of cirrhosis (stage 6) at baseline liver biopsy had significantly higher levels of leptin compared to those with lower fibrosis stage (17436 pg/mL vs 6028.5 pg/mL, p = 0.03). Similar results were observed when the CHC group of patients was analyzed (18014 pg/mL vs 4385 pg/mL, p = 0.05)

In HBV patients significant positive correlations were observed between serum leptin levels and weight (r = 0.65, p = 0.001), BMI and FMI (r = 0.82, p < 0.0001), and in HCV patients between serum leptin levels and age (r = 0.56, p = 0.003) and stage of fibrosis (r = 0.47, p = 0.01). The correlation between serum leptin levels and BMI in healthy subjects was similar to that noted in the patients' group (r = 0.67, p = 0.001).

Significant associations between serum leptin and insulin levels (r = 0.69, p = 0.02) and serum leptin and HOMA-IR (r = 0.61, p = 0.06) were noted in CHC patients infected with genotype 1. Serum insulin levels and HOMA-IR were higher (10.33 vs 5.48 μU/mL, p = 0.03 and 3.02 vs 1.3, p = 0.01 respectively) in HCV patients infected with genotype 1 in comparison with other genotypes, whereas no difference was seen in BMI or leptin levels with respect to genotypes.

A multiple regression analysis, in which the dependent variable was serum leptin levels and independent variables were gender, age, BMI, liver fibrosis and steatosis, transaminase levels, insulin, HOMA-IR and categorized individuals according to their conditions (patients and controls), was performed showing that leptin concentration was jointly significantly correlated with gender, BMI and stage of fibrosis at the baseline liver biopsy in patients with CHB (R^2 ^= 0.6, p = 0.004) and in CHC patients (R^2 ^= 0.72, p = 0.004) .

Age was the only variable significantly associated with cirrhosis; each 10-year increase in age was associated with a 1.7 (95% CI: 1.01–1.14, p = 0.05) fold increase in prevalence of cirrhosis in CHB patients. The same trend [1.7 fold increase in prevalence of cirrhosis with each decade increase in age (95% CI: 0.99–1.15, p = 0.07)] was also observed in CHC patients but statistical significance was not reached.

### 3.3 Factors associated with virologic response to antiviral treatment

We did not observe any significant change of BMI in patients' group (before, at 12 weeks of, and at the end of therapy (26.3, 28.1, 27.8 kg/m^2 ^respectively). Overall (both HCV and HBV patients), 30 of 50 patients (60%) fulfilled our criteria for response (transaminase levels within normal ranges and nondetectable HBV DNA or HCV RNA by PCR). In multivariate logistic regression analysis the only factor negatively associated with response was serum leptin levels. Responders presented significantly lower baseline serum leptin levels than non-responders (4642.5 vs 10733 pg/mL; p-value = 0.002-Table 3).

Our subgroup analysis for HBV patients showed that among the 25 CHB patients, 19 of 25 (76%) experienced an on treatment virologic response after a median of 18.5 months duration of follow-up. Patients with CHB who responded to antiviral treatment had lower serum leptin levels (5334 vs 13111.5 pg/mL; p-value = 0.003) than non-responders. When corrected by gender, the relationship between leptin concentration and lamivudine response was more obvious in the subgroup of male patients with CHB; responders to lamivudine treatment had lower serum leptin levels (4980 vs 13078 pg/mL; p-value = 0.01) than non-responders.

**Figure 1:**
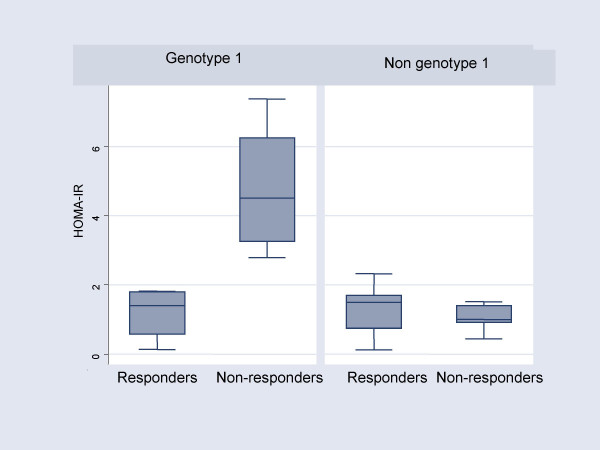
Distribution of homeostasis model assessment for insulin resistance (HOMA-IR) according to sustained virologic response in genotype 1 and non genotype 1 infected CHC patients.

Twenty four of the 25 CHC patients completed full treatment course; one patient discontinued due to severe fatigue and anemia at the 3^rd ^month of treatment. SVR was achieved in 11 of 25 (44%) CHC patients; those CHC patients with baseline HCV RNA higher than 800000 cp/mL were 7.69 (95% CI: 1.08–50, p = 0.04) times less likely to present SVR. After multivariate logistic regression analysis, genotype was the only significant variable associated with SVR; patients infected with genotype 3 were 10.5 (95% CI: 1.5–72.8, p = 0.01) times more likely to present sustained virologic response than non-genotype 3 CHC patients. Subgroup analysis according to genotype has shown that insulin resistance strongly influenced the response only in patients infected with genotype 1 (Figure 1). The rate of SVR was 14% (1/7 patients) in insulin-resistant patients (HOMA-IR > 2), compared 100% (3/3 patients) in patients with HOMA-IR < 2 (χ^2 ^= 0, 01). The SVR rate in genotype 1 patients segregated with respect to HOMA-IR was 100% (3/3 patients) if HOMA-IR < 2, 25% (1/4 patients) if HOMA-IR was between 2 and 4, and 0% (0/3 patients) if HOMA-IR was > 4 (p = 0.04).

## Discussion

The results of this study further confirm that in patients with CVH the physiological correlation among serum leptin level, gender and BMI was well preserved [11,22,23]. In addition, our data have shown that serum leptin levels in patients with HBeAg negative CHB and CHC are associated with severe fibrosis and response to antiviral treatment.

The behavior of leptin concentrations in the course of liver disease due to HBV and HCV infection is still under investigation. Several studies have shown no association between serum leptin and fibrosis in CHB or CHC [4,12,13,24,25]. In contrast to these studies we and others [8,9,26] have shown that serum leptin levels are independently associated with severe fibrosis in patients with CVH. The reason for this discrepancy is not clear. Perhaps the different selection criteria and the different form of chronic infection may explain our results.

The mechanisms involved in the increase of leptin according to the stage of hepatic fibrosis are obscure. The modified stellate cells may express leptin, the cirrhotic liver could be expected to produce leptin and contribute to the rise in circulating leptin with a significant hepatic venous spillover [3]. A cytokine-leptin link hypothesis has also been suggested as a contributing factor to cirrhotic hyperleptinemia; presumably activated tumor necrosis factor-α (TNF-α) in cirrhosis can cause excessive release of leptin from adipose tissue [26]. Henriksen et al. suggested that the elevated circulating leptin in patients with alcoholic cirrhosis was most likely caused by a combination of decreased renal extraction and increased release from subcutaneous abdominal, femoral, gluteal, retroperitoneal, pelvic and upper limb fat tissue areas [27].

Another important finding of our study is that in patients with chronic viral hepatitis, high baseline serum leptin levels represent a negative prognostic factor for response to antiviral therapy. Our subgroup analysis according to viral etiologic factor showed that the above finding was more obvious for male HBV patients. After performing multivariate analysis of several factors low leptin levels were the only feature associated with favorable response to lamivudine monotherapy. It is known that leptin has proinflammatory properties *in vitro*, primarily by upregulating the production of several important proinflammatory Th1 cytokines-including interleukin (IL)-1, IL-6, IL-12 and TNF-α and decreasing the production of the anti-inflammatory cytokine IL-10 [28]. Given the known regulatory effects of these cytokines [29], it thus appears that leptin may help to amplify selected proinflammatory responses; there is evidence that the effects of lamivudine treatment depend on patient's pre-existing active immune response against HBV [30]. It should be noted that our subgroup analysis for CHC patients did not reveal a significant association between serum leptin levels and the response to antiviral therapy. Possible explanations could be the different role of cytokines and immune response in HCV infection and the relatively small number of patients in CHC group; recently Eguchi et al. reported a significant association between high serum leptin level and IFN resistance in a subgroup of 50 patients with low viral load (< 100 MU/L) [31].

Senescence is associated with a progressive increase in insulin resistance [32]. In our study, however, we did not find a significant difference in insulin resistance between patients and controls although the latter group was with higher median age; a reasonable explanation might be the involvement of CVH in the development of insulin resistance, a view that has been also supported by previous reports [33,34].

Recent evidence suggests that insulin resistance is associated with severe fibrosis and poor response to treatment in patients with CHC [35-39]. In our study we determined for first time the insulin resistance in a sizeable cohort of CHB and CHC patients as one could hypothesize an interaction between insulin resistance and leptin regarding their actions in liver fibrogenesis. We did not observe any association in the total group of our patients. However our subgroup analysis has clearly shown an association between insulin resistance and virologic response in patients infected with genotype 1. Our study provides also further evidence that insulin resistance impairs sustained response to antiviral treatment in genotype 1 infected patients and as already proposed by Romero-Gomez et al, HOMA-IR could represent the best host marker to predict SVR in genotype 1 infected patients [38]. Therefore although the number of studies and included patients are small, our data suggest that therapeutic interventions aimed at decreasing insulin resistance in genotype-1 infected patients are necessary in order to increase response rates in these difficult to treat patients.

## Conclusion

Higher leptin levels are associated with severe levels of fibrosis in patients with CVH and represent a negative prognostic factor for virologic response to lamivudine monotherapy in patients with HBeAg negative CHB. Therefore in the forthcoming era of new nucleos(t)ide analogues further clinical research is necessary in order to explore the importance of serum leptin levels in this resistant to conventional antiviral treatment group of patients. On the other hand our data suggest that treatment strategies focusing on improvement of underlying metabolic factors seem appropriate in patients infected with HCV genotype 1.

## Competing interests

The author(s) declare that they have no competing interests.

## Authors' contributions

SM conceived of the study, participated in its design and coordination and revised critically the manuscript, SB participated in the design of the study, made a substantial contribution to acquisition and interpretation of data and drafted the manuscript, CL participated in the design of the study and helped to draft the manuscript, FS participated in the design of the study and performed the statistical analysis, PS made a substantial contribution to acquisition and interpretation of data, AM carried out part of the immunoassays, AC made a substantial contribution to acquisition and interpretation of data, AK made a major contribution to acquisition of data, EV carried out part of the immunoassays, DT participated in the design and coordination of the study and ST made substantial contributions to conception and design of the study, analysis and interpretation of data and have been involved in revising critically the manuscript. All authors read and approved the final manuscript.

## Pre-publication history

The pre-publication history for this paper can be accessed here:



## Figures and Tables

**Table 1:** 

**A**		
Baseline characteristics for men. Data are reported as median and range		
	**Chronic viral hepatitis patients (n = 36)**	**Control Subjects (n = 17)**
**Age (years)**	45.5 (19,73)	62.5 (21,79)
**Height (cm)**	173 (160,190)	173 (167,186)
**Weight (kg)**	81.5 (58,118)	75 (62,100)
**BMI (kg/m**^2^**)**	26.3 (20.3,36.2)	25.1 (21.5, 29.5)
**FMI**	9.99 (5.5, 17.4)	9.09 (6.39, 12.4)
**Glucose (mg/dl)**	96.5 (70,147)*	88 (60,183)
**Insulin (μU/mL)**	7.34 (0.73, 125.8)	9.18 (3.05,17.28)
**HOMA-IR**	2 (0.12–7.36)	2 (0.88–4.89)
**AST (IU/l)**	59 (19,274)*	26 (21,35)
**ALT (IU/l)**	105 (24,366)*	25 (20,36)
**Leptin (pg/ml)**	5134 (695,30430)	5054 (588,15325)

**B**		
Baseline characteristics for women. Data are reported as median and range		
	**Chronic viral hepatitis patients (n = 14)**	**Control Subjects (n = 15)**

**Age (years)**	38 (16,70)	51 (20,74)
**Height (cm)**	165 (156,169)	160 (153,168)
**Weight (kg)**	77 (51,112)	74 (60,94)
**BMI (kg/m**^2^**)**	29.7 (18,39.3)	29.5 (23.1,38.8)
**FMI**	12.6 (3.81,19.7)	12.4 (7.59,17.39)
**Glucose (mg/dl)**	80.5 (70,175)	81 (60,156)
**Insulin (μU/mL)**	5.65 (0.73,16.47)	8.43 (5.67,67.76)
**HOMA-IR**	1.02 (0.12, 6.25)	1.53 (0.95–13.5)
**AST (IU/l)**	55 (38,129)*	27 (20,35)
**ALT (IU/l)**	66 (54,269)*	27 (21,32)
**Leptin (pg/ml)**	11372(1714,43605)	18990(4876,48928)

*p < 0.05 vs controls

**Table 2:** Histological characteristics of the patient population

	**Hepatitis B patients**		**Hepatitis C patients**	
Liver biopsy features	Baseline liver biopsy	Liver Biopsy after treatment	Baseline liver biopsy	Liver Biopsy after treatment
HAI*^1^	8(3,13)*^1^	4 (0,12)*^1^	7 (2,14)*^1^	4 (0,9)*^1^
0–8 N(%)	11(44)	18 (78.26)	16 (64)	10 (77)
9–12 N(%)	13 (52)	5 (21.74)	8 (32)	3 (23)
> 12 N(%)	1 (4)	0	1 (4)	0
Fibrosis	4 (1,6)	2 (0,6)	3 (1,6)	2 (0,6)
Cirrhosis N(%)*^2^	9 (36)	5 (21.74)	5 (20)	3 (23)
Steatosis*^3^	0(0,1)	4 (0,12)	0 (0,3)	0 (0,1)
No steatosis N(%)	16(64)	19 (82.61)	13 (54.17)	12 (92.3)
Mild N(%)	9 (36)	4 (17.39)	10 (41.67)	1(7.69)
Moderate N (%)	0	0	0	0
Severe N (%)	0	0	1 (4.17)	0

*^1^Histological activity (HAI), fibrosis and steatosis data ere expressed as median (range).

*^2 ^Cirrhosis: Stage of fibrosis 5&6 (Ishak classification system)

*^3 ^Steatosis: Mild: < 33% of hepatocytes affected; Moderate: 33% to 66% of hepatocytes affected; Severe: > 66% of hepatocytes affected.

**Table 3:** Baseline characteristics associated with response to antiviral treatment after multivariate logistic regression analysis a) of the whole cohort of patients b) of CHB patients c) of CHC patients (only variables with statistical significance are shown). Data are reported as median and range.

	**Total number of responders (n = 30)**	**Total number of non-responders (n = 20)**	**p-value**
**Leptin (pg/ml)**	4642.5 (917, 32879)*	10733 (695, 43605)	0.002
**CHB patients**	**Responders to lamivudine monotherapy (n = 19)**	**Non-responders to lamivudine monotherapy (n = 6)**	**p-value**
**Leptin (pg/ml)**	5334 (917, 17436)	13111.5 (8844,43605)	0.003
**CHC patients**	**Sustained responders (n = 11)**	**Nonsustained responders (n = 14)**	**p-value**
**Genotype 3**	7 (63.6%)	4/14 (28%)	0.01
